# Case of Neuralgia, in Which the Carbonate of Iron Was Successfully Administered

**Published:** 1826-05

**Authors:** S. D. Broughton

**Affiliations:** Surgeon to His Majesty's 2d Regiment of Life Guards, and to the St. George's and St. James's Dispensary.


*April* 1826.

---

Art. V.—*Case of Neuralgia, in which the Carbonate of Iron was successfully administered.* By S. D. Broughton, Esq. Surgeon to His Majesty's 2d Regiment of Life Guards, and to the St. George's and St. James's Dispensary.

A considerable mass of experience having tended to produce some grounds for confidence in the exhibition of the carbonate of iron in neuralgia; and the nature of this remedy not appearing to offer any objection to its employment, I was induced to make trial of it in the following case.

On the 1st of November, 1823, I was consulted by a lady, residing in the neighbourhood of London, in consequence of her having suffered great pain on the left side of the face during the previous six weeks, the last fortnight of which her malady had been greatly aggravated. Her rest had been disturbed, and she had fallen off in health and appetite, and was much emaciated. She described the pain to be situated chiefly in three distinct places, the temple, the inferior orbital region, and the side of the chin, with a general aching of the left side of the head, and soreness of the integuments of the face covering the parts enumerated. Latterly there had been seldom any suspension of pain altogether, but violent paroxysms succeeded each other rapidly, resembling the shocks of electricity and the stabs of a sharp instrument. Some degree of puffiness about the left eye occasionally appeared. The bowels had been generally irregular, and the tongue was furred.

My first efforts were directed to the digestive organs, and, having cleared the bowels with calomel, salts, and senna, I prescribed forty-five drops of the tinctura opii, to be taken at bed-time; which was followed by a better night's rest than she had long enjoyed. The opiate was repeated on the second night, with similar benefit; but the paroxysms returned in all their violence on the third day. I then determined upon trying the carbonate of iron, keeping up the action of the bowels by moderate aperients, during which the tongue became improved in appearance. I began with half a drachm of the carbonate of iron three times a-day, directing the patient to resort to the opiate as before, if necessary.

On the 8th of November, the patient declared the violence of the pain to have been much reduced; so much so, that the opiate had not been repeated. She had enjoyed some sleep at nights, and was consequently much improved in appearance. The doses of the powder were then doubled.

On the 11th of November, the patient was still going on well. The chief seat of the pain was at the chin, and she declared it to be then bearable. She, therefore, proposed taking the powders twice instead of three times a-day, imagining their effects upon the stomach to be injurious.

The last week in November, she omitted the powders altogether, in consequence of profuse menstruation, accompanied by a furred tongue, loss of appetite, and a sensation of heat at the pit of the stomach. Calomel, magnesia, rhubarb, and ginger, were resorted to with benefit; and the carbonate of iron, in doses of one drachm, was again prescribed, in conjunction with rhubarb and ginger, three times a-day; the original malady having slightly increased.

The powders were continued, though irregularly, throughout December, up to the middle of January. The patient generally felt disposed to omit them when relieved from pain, but immediately resorted to them on its recurrence. About the latter end of January, the stomach seemed to be affected with acidity, when (the neuralgic affection having been subdued,) she left off the powders altogether, substituting soda-water in small draughts, and mild aperients with magnesia.

About the middle of February, the paroxysms of pain returned, violently and in rapid succession at intervals, disturbing her sleep at night. After a dose of calomel, salts, and senna, the opiate draught was renewed, with the same temporary benefit as formerly; the paroxysms returning after the anodyne effects had gone off. The carbonate of iron was then renewed, as prescribed latterly, in doses of one drachm; great attention being paid meanwhile to the state of the digestive organs.

At the end of March, 1824, the powders having been rigidly persevered in, and no paroxysms of pain in the face having returned during three or four weeks, the powders were gradually left off.

Up to the present period (more than two years having elapsed,) this patient has not experienced any symptoms of the neuralgic complaint, under which she laboured periodically for the space of about five months, from the latter end of September, 1823, when she was first attacked, to the beginning of March, 1824, when she returned to society and the enjoyment of her usual health and spirits.

The extent to which the carbonate of iron was prescribed in this case, certainly falls far short of that to which it has been pushed of late; and, from the length of time which the malady took to be entirely subdued, one might, perhaps, reasonably infer that much larger doses, and a more constant perseverance in them, where the stomach will bear such treatment, may produce a quicker termination of the complaint.

The affection above described appeared to be of that genuine neuralgic kind called tic douloureux; and it is remarkable, that neither attention to the digestive organs, which were throughout extremely irritable, nor the use of laudanum, produced any permanent relief; while the administration even of smaller doses than ordinary of the carbonate of iron was invariably followed by the mitigation, and finally by the total removal, of the pain, although it was not without some difficulty that the stomach was at any time capable of receiving the medicine.

I may add, that I have used the carbonate of iron in other cases of neuralgia, both idiopathic and from injury, and with benefit; but the circumstances of these cases were not so strongly marked, and accurately followed up, as this now detailed.

*London; April,* 1826.

